# A Socio-Technical Exploration for Reducing & Mitigating the Risk of Retained Foreign Objects

**DOI:** 10.3390/ijerph15040714

**Published:** 2018-04-10

**Authors:** Siobhán Corrigan, Alison Kay, Katie O’Byrne, Dubhfeasa Slattery, Sharon Sheehan, Nick McDonald, David Smyth, Ken Mealy, Sam Cromie

**Affiliations:** 1 School of Psychology, Trinity College Dublin, Dublin 2, Ireland; kayam@tcd.ie (A.K.); obyrnekj@tcd.ie (K.O.); nmcdonld@tcd.ie (N.M.); sdcromie@tcd.ie (S.C.); 2 Faculty of Medicine and Health Sciences, Royal College of Surgeons (RSCI) in Ireland, Dublin 2, Ireland; dubhfeasaslattery@rcsi.ie; 3 Medical Professionalism, RCSI and Bons Secours Health System, Dublin 2, Ireland; 4 Coombe Women and Infants University Hospital, Dublin 8, Ireland; ssheehan@coombe.ie; 5 Department of Surgery & Clinical Director Perioperative Services, University Hospital Waterford, X91 ER8E Waterford, Ireland; david-zxc@hotmail.com; 6 Department of Surgery, Royal College of Surgeons (RSCI) in Ireland, Dublin 2, Ireland; kmealy@rcsi.ie

**Keywords:** retained foreign objects, patient safety, human factors, sociotechnical systems, risk management, multi-disciplinary approach, process modelling

## Abstract

A Retained Foreign Object (RFO) is a fairly infrequent but serious adverse event. An accurate rate of RFOs is difficult to establish due to underreporting but it has been estimated that incidences range between 1/1000 and 1/19,000 procedures. The cost of a RFO incident may be substantial and three-fold: (i) the cost to the patient of physical and/or psychological harm; (ii) the reputational cost to an institution and/or healthcare provider; and (iii) the financial cost to the taxpayer in the event of a legal claim. This Health Research Board-funded project aims to analyse and understand the problem of RFOs in surgical and maternity settings in Ireland and develop hospital-specific foreign object management processes and implementation roadmaps. This project will deploy an integrated evidence-based assessment methodology for social-technical modelling (Supply, Context, Organising, Process & Effects/ SCOPE Analysis Cube) and bow tie methodologies that focuses on managing the risks in effectively implementing and sustaining change. It comprises a multi-phase research approach that involves active and ongoing collaboration with clinical and other healthcare staff through each phase of the research. The specific objective of this paper is to present the methodological approach and outline the potential to produce generalisable results which could be applied to other health-related issues.

## 1. Introduction

Unintentionally retained foreign objects (RFOs) are considered to be a ‘never event’ in healthcare, that is, theoretically preventable [1]. Many incidents of RFOs are comparatively small [2] and may not be discovered until many years after the actual procedure. This coupled with the fact that much research in this area is retrospective and anecdotal makes it very difficult to establish an exact incident rate. Therefore, defining the real burden of RFOs in the clinical setting is challenging [3], but it is widely accepted that it comprises very real negative impacts. These include negative impacts on:

- Patients: Symptoms experienced by patients vary depending on the nature of the RFO (inert or not), the duration it has been left in situ and the location in which it has been left. Symptoms identified in a recent national, 10-year review of closed claims pertaining to RFO in maternity and gynaecology services (performed by [4] at the State Claims Agency) included pain (acute and chronic), foul smelling discharge, difficulty walking, vaginal bleeding and general malaise. Complications included weight gain, hirsutism, pelvic inflammatory disease, infertility and the need for further surgery. Medium- and long-term associations were identified and diagnosed by both patient and consultant physician respectively on over one-third of patients with RFO in gynaecology services in this review. Problems included depression (requiring psychotherapy and pharmacotherapy), mild adjustment disorder and anxiety regarding further surgery and return to work. RFOs and their sequelae often culminate in a loss of trust in the medical profession.
- Financial costs: There is a varied cost implication depending on location and the impact it has on the individual affected. In a study conducted by [5], the variance in cost of a RFO ranged from between $51 to $3,988,829 with an average cost for legal practice payments of $86,247 alone.
- Reputational damage at both the individual clinician level and at the institutional level. Stawicki et al. identifies that RFOs are in the main a result of team- or systems-based errors as opposed to an individual human error [6]. However, as a result of the ‘blame’ culture that exists within the healthcare industry this perception leads to a tendency of providers to attempt to conceal the error or shift blame away from them [7].

There are many studies that identify varied incident rates, based on these rates [8] estimates the incidence rate to range from 1/1000 to 1/19,000. A number of U.S.-based research studies have estimated the rate of RFOs to be from between 1/1000 to 1/1500 in abdominal cases where the majority of RFOs were sponges/swabs. In most of these a correct swab/sponge count had been reported. From an Irish perspective, approximately 200 clinical incidents relating to RFOs are reported annually to the Irish State Claims Agency (SCA) (data retrieved from [9]). Between the years from 2011 to 2015, over 1000 such incidents were reported to the SCA. Primarily these involved surgery, medicine, maternity and gynecology services. In particular, surgery and maternity care are of most concern due to the larger number and cost of claims.

Two of the key limitations regarding the research on RFOs are the almost exclusive focus on surgical settings where two-person swab/sponge counts are routine and the lack of empirical and comparative research. Therefore, while generic principles and interventions for addressing the problem have been developed [10,11], it is imperative to adapt these to specific cultural contexts and to both surgery and the Delivery Suite. Very few studies (e.g., [12]) make reference to the practice in the Delivery Suite and it is unclear if the practices in place in the operating room are applied to the Delivery Suite/Labour Ward.

A critical first step to addressing the problem of RFOs is identifying key risk factors. The Joint Commission provided an analysis of the most common risk factors of RFO events reported to them [13]:

- Absence of policies and procedures
- Failure to comply with existing policies and procedures
- Problems with hierarchy and intimidation
- Failure in communication with physicians
- Failure of staff to communicate relevant patient information
- Inadequate or incomplete education of staff

Many interventions have been utilised to prevent foreign objects from being retained. These strategies include counting and cavity sweep protocols, use of radiography or adjunct technologies, education and training [14]. Observations, process mapping and focus group sessions conducted by [15] identified potential failure points and their causes during surgical swab/sponge management. The most common causes of count failures included distraction, multitasking, procedure non-compliance and time pressure. Using the Healthcare Failure Mode and Effect Analysis (HFMEA), severity and probability of occurrence were rated and analysed to determine each failure’s significance. The significance of these failures was rated as catastrophic (potential harm or death). Core recommendations included the introduction of an additional time-out within the closing counts and the use of assistive technology.

Relying on manual counts alone has been seen as ineffective; [16] recorded the sensitivity of manual counting as 77% and specificity as 99%. However, attempts to improve these results have been seen with the introduction and implementation of standardised counting strategies [17]. It is clear that the need for additional measures in conjunction to manual counts is required and many international guidelines (i.e., Association of periOperative Registered Nurses (AORN), the Joint Commission) recommend the evaluation of adjunct technologies. These include intraoperative radiography, bar-coding, radiofrequency detection (RFD) and radiofrequency identification technology (RFID) [13,14]. In a previous study performed by [18], using intraoperative radiography to verify the absence of a retained swab/sponge showed only 67% (12) of RFOs identified with the use of X-ray.

The first systematic evaluation of the sensitivity of RFD in a prospective cross-over study including the risk factor; increased BMI showed RFD systems scoring 100% for both specificity and sensitivity [19]. Evaluations of the effectiveness of a radiofrequency detection system in addition to manual counts were performed over a 10-month period [3]. This system aided the resolution of a near-miss event and enhanced identification of misplaced swabs with no incidence of retained sponges. Circulating nurses completed post-operative surveys with 95% reporting improved confidence in counts using the RFD system. In addition to this is a 5-year prospective study evaluated the process of mitigating retained sponges using an RFD system; there were no retained sponges and 11 near misses were identified by the RFD system [20]. In two cases, the RFD system identified sponges in which a correct count was reported before closure, thus counting alone is unreliable [20].

This level of analysis alone is insufficient to provide us with an understanding of what actually happens in normal operational practice and thus our ability to design effective interventions is curtailed. It may inform us when RFOs are more likely to happen, but it does not suggest how or why they occur. For instance, it makes sense that an incorrect surgical count would be a risk factor, but how and why do incorrect surgical counts happen? What are the key differences between surgical settings and maternity Delivery Suite/Labour Ward settings? This is where a Socio-Technical Systems (STS) analysis and modelling of the human and organisational factors is critical, especially in relation to implementing effective change.

A consistent finding in articles on quality improvement in healthcare is that change is difficult to achieve [21,22]. Much of the weakness in the research literature is due to a failure to develop the interventions systematically, using best available evidence and appropriate theory. While there are many examples of successful interventions, there are also numerous safety and quality improvement interventions that have failed to have an impact—particularly in terms of bringing about long-term behavioural change and improvements in patient safety.

### 1.1. Objectives

Therefore, the overall objectives of this HRB-funded research are as follows:

- Analyse and understand the problem of RFOs in surgical and maternity settings in Ireland
- Develop hospital-specific RFO management processes and implementation roadmaps
- Provide RFO management toolkits
- Ensure impact in healthcare and academia

The specific objective of this paper is to present the proposed methodological approach for the research. The overall approach has been adapted from collaborative human factors-based research in other safety critical industries (most notably aviation and emergency services) focusing on managing the risks in effectively implementing and sustaining change. Therefore, this approach focuses on new methodologies in implementation of health systems interventions that involve active and ongoing collaboration with clinical and other healthcare staff through each phase of the research.

### 1.2. New methods for Human Factors Socio-Technical Modelling

Interest in the Socio-Technical Systems (STS) approach to understanding complex safety systems reflects a growing belief that many dimensions of safety are emergent properties of such systems [23]. A STS is the synergetic interaction and integration of humans, processes, information and knowledge flows, technology, structures and the external environment in the workplace. Interactions are key in the STS approach and recognising the broad STS and the respective interactions between the different levels contribute to a more effective and integrated analysis of the current operational practice [24].

Functional Resonance Analysis Method (FRAM) [25] and Systems-Theoretic Accident Model and Processes (STAMP) [26] represent the current state-of-the-art for social-technical modelling. Both the FRAM and STAMP approach to modelling and enquiry is retrospective and largely atheoretical—trying to find the best representation of the complexities of a particular case as it happened in the past. There are two fundamental limitations of these approaches:

- It is impossible to generalise from one case analysis to other potential situations.
- When one uses FRAM or STAMP to analyse a particular case in order to project how it might pan out in the future, it is necessary to put values on key variables in order to draw inferences about how they might perform and influence other variables in the future [27]. In the absence of a theory, this becomes rather subjective and can easily lead to wrong predictions. This is a serious problem that is not commonly recognised by the advocates of these models [27].

This project will deploy an integrated evidence-based assessment methodology for social-technical modelling (SCOPE Analysis Cube) and bow tie methodologies that has been developed based on sound theoretical principles and rigorously tested and validated (EU-Funded projects MASCA—Managing System Change in Aviation, PROSPERO—Proactive Safety Performance for Operations, ACROSS—Advanced Cockpit for the Reduction of Stress and Workload [28,29,30,31,32]). The core theoretical proposition of this research methodology is to focus on the functionality and interactions of the current STS which are key to understanding it more effectively, changing it to achieve better outcomes and an improved functioning for a safer future system.

This research will focus on four interlocking and interdependent levels of analysis: (i) process functionality (operational processes and management processes); (ii) social cohesion (trust, social networks); (iii) technology, all of which are mediated through; (iv) collective knowledge and information cycles. Therefore, the overall methodology focuses on an analysis and dynamic process modelling at each of these four levels.

In each of these elements the operational, human and social system can be mapped and analysed according to each logic.

1. A process map details the sequence involved in transforming input to output. This supports a high-level ‘decision point map’ which can both represent the status of the relevant dependencies, and show how the dependencies of intersecting processes can be represented (e.g., map the key steps in the delivery process and highlight the current barriers/blockers).
2. An information map denotes the flow of information and the sharing of knowledge. It is designed to highlight cycles of transformation and validation of knowledge and information (e.g., what type of patient information is required in what format, etc.).
3. A social relations/team map describes the network of connections between people involved in the process. This highlights the reciprocal relations amongst sub-groups which provide social structure to sustain activity (e.g., capturing the lived experiences and challenges of different healthcare staff) [27].

**Figure 1:**
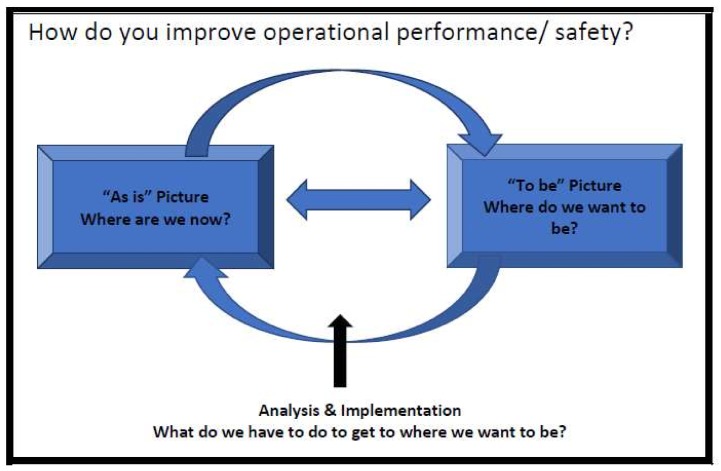
From “As is” to “To be” (adapted from [27]).

As well as modelling the relevant surgical and maternity services processes, the project will also model the ‘as-is’ and ‘to-be’ risk management strategy using a bow tie analysis. A bow tie analysis is a method of analysing risk and depicting how it is, or could be managed in a particular context. It derives its name from the shape of the depiction—like a bow tie. The hazard and risks identified using SCOPE Analysis Cube are therefore further examined to produce a picture of risk within the operational process. In essence, a moving bow tie along the operational process (at selected points along the operational process) is obtained. The value of this approach is that you gain an insight into the risk at a particular point in time and can address “how” change must be effected in order to move from the “As is” in relation to RFO in both surgery and maternity services and proposing interventions to meet the needs (the ‘to be’ situation) to reduce and mitigate the risk of RFOs operations (Figure 1).

In addition to improved capturing of operational reality, rich process models also function as an effective means of developing a common operational picture (COP) between stakeholders. Process stakeholders sometimes function with diverse understandings of a process—its objectives, critical tasks, etc. Developing a COP helps to enhance collaboration through a shared understanding of the process.

### 1.3. Context of the Research

This study will involve participants from two Irish hospitals—the Coombe Women and Infants University Hospital (maternity services) and University Hospital Waterford (surgery). Participants will be involved in interviews, take part in focus groups and participate in observational studies to assist in defining and addressing the problems and risk factors associated with RFO. This will involve pilot and implementation phases for a RFO management process within the hospital setting. Implementation frameworks will include introducing, customising and embedding the process into the pilot hospitals.

## 2. Materials and Methods

This research proposal is framed within the development and pilot stage of the UK Medical Research Council’s key elements of developing and evaluating a complex intervention [33].

Statement on ethical approval: Favourable ethical opinion for the research has been obtained from Trinity College Dublin, Coombe Women and Infants University Hospital and University Hospital Waterford.

Two key features of their recommendations include:

- “The process of developing and evaluating a complex intervention has several phases, although they may not follow a linear sequence”. The research in the project covers the development and pilot phases.
- “Complex interventions may work best if tailored to local circumstances rather than being completely standardised”. This research will define complex interventions but also builds a customisation and implementation process.

The overall research therefore involves a multi-disciplinary, multi-phase approach actively involving end users at each phase. Involving the key stakeholders in the overall research process increases the likelihood of the identification of a feasible, acceptable, effective and sustainable intervention. It is recognised that RFO is an issue across many domains of healthcare. It is not possible, however, to design, develop and pilot an intervention appropriate for every domain for the proposed research. As such, after careful consideration and conversations with key stakeholders it was decided to focus the proposed research on surgery and maternity services.

- Phase 1: Adapting SCOPE Analysis Cube to the specific research objectives topic
- Phase 2: Developing process maps & STS analysis
- Phase 3: Examining the Risk in Proposed Change Interventions (Bow Tie Analysis)
- Phase 4: Selecting Interventions & Implementation Road Map

### 2.1. Phase 1: Adapting SCOPE Analysis Cube to the Specific Research Objectives Topic

The framework for compiling the interacting Process Models in this research is based on the operational framework and the SCOPE Analysis Cube methodology [27] that was developed and tested largely within the aviation domain [32].

**Figure 2:**
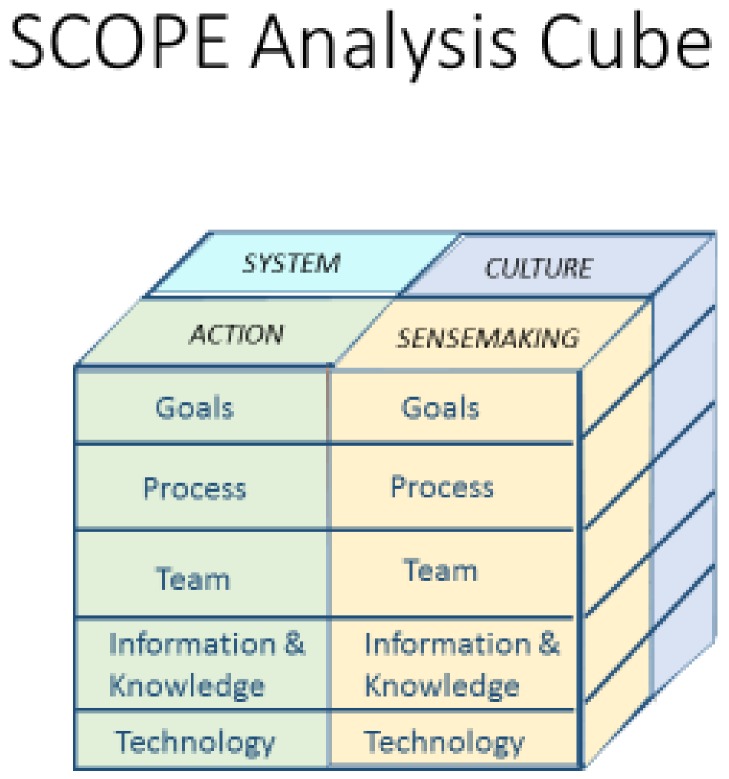
SCOPE Analysis Cube Adapted from [34].

The SCOPE analysis cube is used to structure the analysis in a systematic manner. This is used for both the “As is” and “To be” analyses. In order to break a large socio-technical system down into smaller and more digestible chunks, it can be interrogated from four specific angles: System, Action, Culture and Sense-making (see Figure 2). A research methods workshop was conducted with a sample of end users in order to present the SCOPE analysis cube and contextualise it in relation to healthcare. The following section provides a step-by-step guide of the various elements of the cube.

The “System” refers to the functional relationships within the socio-technical system. The “Action” refers to what happens in the system that can be measured. “Culture refers to the shared meanings, norms & values of the organisation and its members. “Sense-making” refers to how the system works and what needs to be done from each stakeholder’s/end user’s perspective. Each of these four is further examined under the structure of goals, process, team, information & knowledge, technology. An example of each is presented in the following tables (Table 1, Table 2, Table 3 and Table 4): k System.

Goals: what are the outcomes, products or services involved?

Process: What resources, tasks, critical points, dependencies are involved?

Team: Who is in the team? How do they get on? Who is accountable?

Information & Knowledge: How is data transformed? How does the knowledge cycle work?

Technology: What are the relevant technology functions? How does automation support those involved?

Action

Goals: what are the key performance indicators and risks involved?

Process: What types of variability, uncertainty, what hazards are involved?

Team: What level of co-ordination is there? Who is the co-ordinator? How is this done?

Information & Knowledge: What operational data are used? How are they used, gathered?

Technology: What are the relevant technology functions? How does automation support the operational process?

Sense-making

Goals: what are your objectives?

Process: What aspects of workload and error potential are involved? How are these measured/monitored?

Team: How do people collaborate? What is the Team structure? How are people supported? Do they Trust one another?

Information & Knowledge: How do people anticipate? Do they do this well? Is there good situation awareness? What levels of decision support are available? How is “Know-how” captured?

Technology: How does the Human Machine Interface (HMI) support those involved?

Culture

Goals: What are the inherent values within your organisation? Your team?

Process: What are the cultural norms within your organisation? Your team?

Team: Are there sub-cultures within your organisation? Your team? How do they contribute to or detract from the overall successful outcomes of your day-to-day working practices?

Information & Knowledge: What is the partially shared collective understanding of your team? Your organisation?

Technology: What artefacts/technologies are used? What is state-of-the-art technology in your field? How does this/would this contribute to successful working operations?

Based on the SCOPE Cube Analysis, an interview protocol was designed providing a set of questions driving a further analysis of each of the elements. This is not a simple question and answer process that follows a rigid structure through each of the elements. The researcher is meant to tailor the enquiry to what is relevant to the participant’s experience at the current stage and facilitate participant stories and narratives.

These semi-structured interviews will be supplemented by a systematic analysis of relevant documentation. See Table 5 for a list of relevant documentation. It is an attempt to build up a dossier of knowledge about the current ‘as is’ situation and acceptability and usability of any new proposed interventions. Such dossiers should be powerful enough to drive recommendations that are sufficiently cogent and authoritative to influence the overall development and implementation plan and ensure acceptability and trust while demonstrating the value of proposed interventions. This interview protocol also provides a substructure for developing focus group research activities with subject matter experts and end users.

### 2.2. Phase 2: Developing Process Maps and STS Analysis

In order to build up this picture of the current ‘as is’ situation and develop process maps of both the maternity and surgical processes, semi-structured interviews (as developed in phase 1) will be conducted with the following sample (see Table 6 and Table 7):

Surgery: (Aim: 20 participants)
Maternity: (Aim: 20 participants)

**Figure 3:**
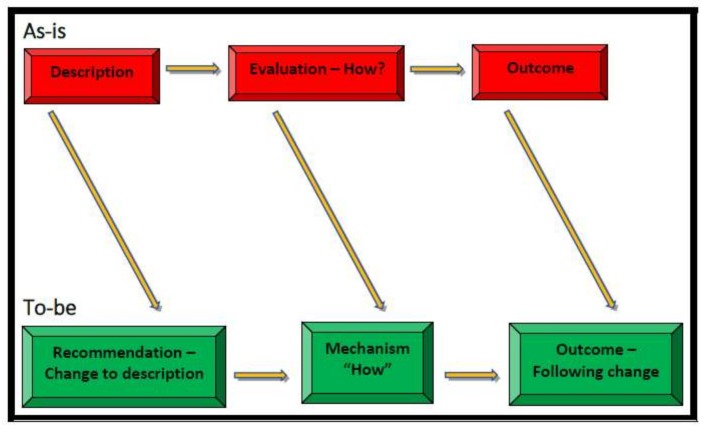
Gap Analysis (between “as is” and “to be” Adapted from [35], p. 31).

Following the interviews, the SCOPE Analysis cube will be populated in order to present the current situation and this will be supplemented by process maps at each of the levels (Process, Knowledge/Information and Team). The next stage in the methodology is to run at least three validation focus groups. One in each of the hospitals and one with subject matter experts (the project’s scientific advisory group that is made up of academics, clinicians and patient advocates) in order to present and validate the findings and populate the Cube for the ‘to be’ situation. Once both conditions have been analysed and agreed, the next step involves conducting a gap analysis between the ‘as is’ and the ‘to be’ situation and to establish the recommended practices and roadmap for mitigating Retained Foreign Object. Hence, in Figure 3 the “To be” picture has changed from Description to Recommendation.

A further validation will be carried out through a comparison of data with findings from an analysis of closed claims from the State Claims Agency.

### 2.3. Phase 3: Examining the Risk in Proposed Change Interventions (Bow Tie Analysis)

**Figure 4:**
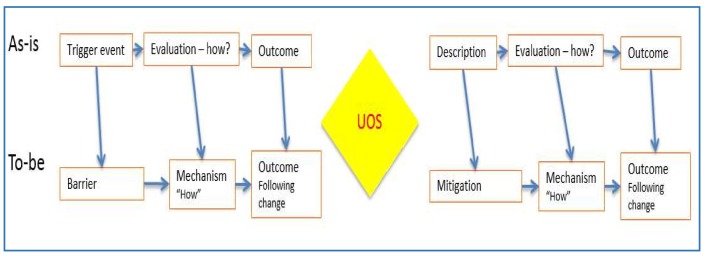
Structure of a global bow-tie analysis (adapted from [35], p. 33).

**Figure 5:**
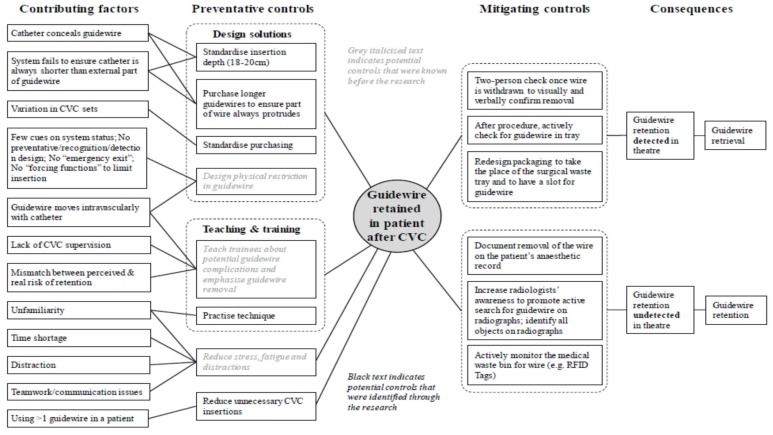
Bow-tie diagram showing contributing factors, controls and consequences behind an event involving a retained guidewire [37], (CVC—Central Venous Catheterisation, RFID—Radio-frequency Identification).

As well as modelling the relevant surgical or maternity services processes, the project will also model the ‘as-is’ and ‘to-be’ risk management strategy using a bow-tie analysis. At the centre of the bow-tie is the “top event” of interest and, in this case, the RFO. To the left are the sequences of events that could lead to this top event (e.g., error in counting instruments) and to the right are events that could follow from this event (e.g., patient discomfort). When these events are elaborated on, the picture can be augmented by inserting risk controls that are, or could be, used to prevent the top event (e.g., using tailed swabs, or mitigate it (e.g., X-raying the patient after surgery). See Figure 4 and Figure 5 for examples of bow tie analyses.

The SCOPE Cube Analysis has been extended to employ a bow tie analysis using the general framework of the Aviation Risk Management Solutions (ARMS) bow-tie methodology. The ARMS is increasingly regarded as a standard for aviation operational risk assessment [36].

Therefore, the integration of these methodologies has the benefit that, as well as effectively analysing the process and risk, it provides a graphical representation that can help communicate the methodologies and develop a shared understanding. By employing both of these methodologies, it is possible to develop a “common operational picture” of how risk is managed now and how it should be managed in the future. The “how” and “when” and “where” of risk reduction measures can accurately and effectively be put in place [32].

A focus group consisting of key stakeholders from both hospitals and subject matter experts (project advisory group) will be conducted in order to present and validate the overall bow tie analysis.

### 2.4. Phase 4: Select Interventions and Implementation Road Map

The implementation road map will define the optimal steps to promote effective customisation, adoption and implementation of the proposed new intervention(s) in both surgical and maternity settings across both of the hospitals. It will include recommendations for stakeholder engagement, training and promotion of the process, getting buy-in from management and procurement (for purchasing of optimal equipment), monitoring and adjustment of the process. At the sector level it will include recommendations for enhancing RFO management training in undergraduate and postgraduate medical and nursing curricula, ensuring the effective reporting, investigation and analysis of RFO cases and just culture policies to promote an open culture in relation to RFO.

## 3. Discussion

‘Never events’ are typically rare but can lead to serious adverse outcomes in healthcare. They are perceived to be preventable, and include the retention of foreign objects in a patient’s body. This research programme aims to explore this problem by applying an integrated suite of methodologies that not only focuses on the ‘human error’ but also at a system level in order to establish an effective RFO management process and an implementation roadmap. The key focus of the research moves to the implementation of the process rather than the process itself, which is why it is an advancement from other research studies.

This research will likely have multiple impacts on the clinical care programmes within the two hospitals and in terms of patients and healthcare at both the national and international levels.

- RFO as an issue, traverses multiple specialties from surgery, maternity, gynaecology to medicine. Identifying a successful model of implementation of human factors risk management in healthcare which traverses multiple disciplines and multiple specialties would be a significant progressive step. Additionally, it would fully support the Health Service Executive’s (HSE) Integrated Care aims through the Five Integrated Care Programmes of which maternity is one. Poor communication, hierarchical structures and the absence of a culture of “speaking up” are identified barriers to preventing change and improving care.
- Process and outcome measures will be identified to support ongoing evaluation of RFO defined as a “serious reportable event” (SRE) by the HSE [38]. RFO is classified as an event which should never happen and considered “unacceptable and eminently preventable” by the National Health Service (NHS) [39].
- This research project may lead to reduced physical and psychological harm to patients and reduce risk of mortality. While deaths from RFO have not been recorded in the Irish Health Service in recent times, the frequency of RFO incidents makes them a significant risk in this regard. Cases of septicemia related to RFO have been identified which required urgent admission to hospital, intravenous antibiotics and surgical removal of RFO. Additionally, reduction in RFO will reduce the risk of “second victim” (which may include symptoms of anxiety, stress, loss of confidence, insomnia, depression and sometimes suicide) in clinicians involved in a RFO incident.
- Improved public confidence and trust in the national healthcare system may stem from this research work through prevention of incidents of RFO. The latter have the ability to attract significant negative media attention and, together with other SREs, are used as an international comparator for healthcare systems. Claims have a reputational cost to the Irish Healthcare System at an international, national, institutional and individual healthcare professional level.

Reputational cost to the Irish Healthcare System at an international, national, institution and individual healthcare professional level. RFOs are considered as medical negligence. They have the ability to attract significant negative media attention. They are one of the listed Serious Reportable Events which are used as international comparators for healthcare systems. Internationally, a RFO is identified as a “never event” and the Centres for Medicare and Medicade Services (USA insurance companies) do not reimburse hospitals to manage this event which they feel “should never happen“. Prevention of these events would prevent significant reputational damage to the hospital and rebuild trust in the Irish Healthcare System. RFOs are also likely to result in adverse sequelae for the healthcare professional, including psychological trauma like other adverse events, and may lead to the development of symptoms consistent with the “second victim”.

Although the focus of this research project is on RFO, the methodological approach could be applied to any aspect of health systems research that involves the interplay of people, processes, knowledge/information and technology. The underlining theory and methods proposed in this paper are based on over twenty years of continuous research in complex, real world safety critical industries. This focus on the functionality and interplay of a current STS system is key to understanding normal operational practice (i.e., what is working well as well as what is not working optimally), managing the inherent risks in the system and changing it to deliver better outcomes. Therefore, regardless of the healthcare system under study, our research would indicate that healthcare systems need to be viewed as complex adaptive systems. The key aspect here are the interactions of the various components and that they cannot be viewed in their individual capacity alone, nor in organisational silos. Research by Sittig and Singh on the adaption of socio-technical systems thinking to Health Information Technology (HIT) found that the most important result is that ‘’hierarchical decomposition (i.e., breaking a complex system, process, or device down into its components, studying them, and then integrating the results in an attempt to understand how the complete system functions) cannot be used to study HIT’’ [40] (p. 69). Kamal et al. highlight the fundamental necessity for a contextualised systems approach especially when dealing with Big Data [41]. This research methodology advocates such a perspective.

The approach proposed in this paper also advocates that sets of independent elements (e.g., process, information flows, etc.) cannot be studied in isolation and then consolidated to develop a picture of the future ‘to be’ situation. It is in the understanding of how the various elements interact and how interdependent they are on each another. They must be understood and mapped as ‘’multiple, interacting components with non-linear, emergent, dynamic behavior (i.e., small changes in one aspect of the system lead to small changes in other parts of the system under some conditions, but large changes at other times) that often appears random or chaotic’’ [40] (p. 70). We would argue that this is very typical of complex health systems, and the approach proposed in this paper reflects these interactions.

One limitation is that the proposed methodology has been largely developed and tested within the aviation sector. While the health sector can learn from safety research within the aviation domain, the lessons learned are not all seamlessly transferrable. Therefore, this project provides the opportunity to apply this methodological approach in the first instance in RFO and then to further understanding and enhancing healthcare systems.

## 4. Conclusions

This research has the potential to produce generalisable results which could be applied to other issues and/or situations outside the specific area outlined in this application. The approach taken in this project is specifically designed not just to be generalizable but to generate tools to enable this generalisation. It starts from the premise that the solution to RFO is not a method or even a set of methods—it is the integration of methods in a process that is customised for the context and effectively implemented in that context. The RFO management process will be deliberately designed as a generic process to be tailored to the specific context and the implementation roadmap will include the customisation tool. The results will thus be ideal for extension to other procedures where RFO can be problematic (e.g., catheter guidewires in cardiology), and to other hospitals. Once successful in the two pilot hospitals, this project has the potential to be spread across surgical services in all acute hospitals nationally, all 19 maternity services nationally and be extended to international use. In addition, the approach to managing human factor risks in surgery—through process analysis, bow tie analysis and implementation roadmaps—can be adapted to other challenges such as wrong side or site surgery and medication errors.

## Figures and Tables

**Table 1:** Example of “System”.

Goals	Best Care for Each Patient—Best Outcomes & Experiences, Reduction in Adverse Outcomes
Process	What tools, equipment, resources (including time and people), are available? What tasks are relevant? What are the critical points for the process? What are the relevant dependencies?
Team	Team relations, accountability, how do people collaborate? Is there a time, place mechanism for them to talk?
Information & Knowledge	Data transformation, knowledge cycle. Do people get the right information at the right time?
Technology	Technology functions/automation. How does software (& hardware) support the operational process? How do they support the people?

**Table 2:** An example of “Action”.

Goals	Reduction in Adverse Outcomes
Process	All relevant tasks (ward, theatre, admin) mapped (including variability, uncertainty, hazards)
Team	Co-ordination. Are everyone’s responsibilities clear? Do they know what they should be doing (with whom? And when?)?
Information & Knowledge	Operational data Information is captured AND shared between the right people at the right time
Technology	Automation Centralisation, standardisation of technology where appropriate.

**Table 3:** An example of “Sense-Making”.

Goals	Shared Understanding of Roles and Responsibilities for All Operations (Medical, Surgical, Admin, etc.)
Process	Staff understand the operational process AND what that means in terms of workload, potential risks, hazards, error.
Team	Collaboration—Does everyone have an opportunity to contribute? Team structure & support (awareness of social network) Trust—Is there transparency in the process? Everyone’s roles and responsibilities?
Information & Knowledge	Are staff able to anticipate what is happening/should happen? Is there good (individual and collective) situation awareness? What decision support is available for staff to use (independently and as a group)?
Technology	Does the software and interface support the flow of information as it should for individuals, teams, management?

**Table 4:** An example of “Culture”.

Goals	Best Care for Patient Best Care for STAFF Shared Vision for Above Valued by Staff
Process	What norms of behaviour & everyday practice? What are the relevant organisational routines?
Team	How is/are the organisation(s) divided? Are there professional/personal subcultures? Would it be useful to provide an analysis of this?
Information & Knowledge	Good common operational picture, shared situational awareness and understanding. Flow of information supports this.
Technology	Technology (state-of-the-art) is trusted by staff, used by staff and designed/reviewed by staff (sense of ownership)

**Table 5:** Relevant documentation.

	Hospital Information/Documentation
1.	Prevention of Retained Surgical Items Policy
2.	Swabs, Sharps & Instrument Count Policy
4.	Count sheet (in-chart documentation used for every procedure)
5.	Post-operative note (in-chart Surgeon’s operative sheet)
6.	C.S.S.D./H.S.S.D. (Central Sterile Services Department/Hospital Sterile Services Department) Instrument Policy (sterile instrument service)
7.	Reporting Protocol/Policy
8.	Incident Report Form/Risk Management Form
9.	Surgical Safety Checklist Policy/Documentation
10.	Policy availability—Hard copy or Intranet
11.	Monitoring process for read policies
12.	New staff orientation information on Foreign Object Retention

**Table 6:** Target participant sample surgery.

Profession	Role	Number
Surgeon	Consultant	2
	Registrar	2
	Senior House Officer	1
Nurse	Scrub	3
	Circulating	3 (total = 6)
Anaesthetist	Consultant	1
	Registrar	1
	Senior House Officer	1
Clinical Nurse Manager (CNM)	CNM 3	1
	CNM 2	1
	CNM 1	(total = 2/3)
Clinical Facilitator	Policy involvement	1
Clinical Risk Manager	Risk manager	1
Stores Manager	Equipment/stock orders	1
Cleaners Sterile Services	Theatre cleaner	1
	Equipment sterilisation	1

**Table 7:** Target participant sample.

Profession	Role	Number
Obstetrician	Consultant	2
	Registrar	2
Midwife/Nurse	Scrub/Circulating (theatre)	2
	Midwife	6
Anaesthetist	Consultant	1
	Registrar	1
	Senior House Officer	1
Clinical Midwife/Nurse Manager (CMM/CNM)	CMM/CNM 3	1
	CMM 2	1
	CMM 1	1
Clinical Facilitator	Policy involvement	1
Clinical Risk Manager	Risk manager	1
Stores Manager	Equipment/stock orders	1
Cleaners Sterile Services	Theatre cleaner	1
	Equipment sterilisation	1
